# Mucosal immunity in COVID-19: a comprehensive review

**DOI:** 10.3389/fimmu.2024.1433452

**Published:** 2024-08-14

**Authors:** Saeed Awad M. Alqahtani

**Affiliations:** Basic Medical Sciences Department, Faculty of Medicine, Taibah University, Medina, Saudi Arabia

**Keywords:** mucosal immunity, immune evasion, SARS-CoV-2, COVID-19, vaccines

## Abstract

Mucosal immunity plays a crucial role in defending against coronaviruses, particularly at respiratory sites, serving as the first line of defense against viral invasion and replication. Coronaviruses have developed various immune evasion strategies at the mucosal immune system, hindering the recognition of infected cells and evading antibody responses. Understanding the immune mechanisms and responses is crucial for developing effective vaccines and therapeutics against coronaviruses. The role of mucosal immunity in COVID-19 is significant, influencing both local and systemic immune responses to the virus. Although most clinical studies focus on antibodies and cellular immunity in peripheral blood, mucosal immune responses in the respiratory tract play a key role in the early restriction of viral replication and the clearance of SARS-CoV-2. Identification of mucosal biomarkers associated with viral clearance will allow monitoring of infection-induced immunity. Mucosally delivered vaccines and those under clinical trials are being compared and contrasted to understand their effectiveness in inducing mucosal immunity against coronaviruses. A greater understanding of lung tissue-based immunity may lead to improved diagnostic and prognostic procedures and novel treatment strategies aimed at reducing the disease burden of community-acquired pneumonia, avoiding the systemic manifestations of infection and excess morbidity and mortality. This comprehensive review article outlines the current evidence about the role of mucosal immune responses in the clearance of SARS-CoV-2 infection, as well as potential mucosal mechanisms of protection against (re-)infection. It also proposes that there is a significant role for mucosal immunity and for secretory as well as circulating IgA antibodies in COVID-19, and that it is important to elucidate this in order to comprehend especially the asymptomatic and mild states of the infection, which appear to account for the majority of cases. Moreover, it is possible that mucosal immunity can be exploited for beneficial diagnostic, therapeutic, or prophylactic purposes. The findings from recent studies on mucosal immunity in COVID-19 can be used to develop effective vaccines and treatments that can effectively target both mucosal and systemic immune responses.

## Introduction

Mucosal immunity is often overlooked but plays a crucial role in defending against respiratory pathogens like severe acute respiratory syndrome coronavirus 2 (SARS-CoV-2) (1). This aspect of the immune system protects the primary entry points for infections, the mucosal surfaces (2). Given that SARS-CoV-2 primarily infects the upper respiratory tract, an effective immune response is needed at the respiratory mucosal surfaces. Yet, most research has focused on serum antibodies and systemic cell-mediated immunity, overlooking the significance of mucosal immunity (3). However, recent evidence suggests that mucosal immunity, particularly secretory IgA antibodies, is important in the asymptomatic and mild cases of COVID-19, which constitutes most cases (3). Understanding mucosal immunity is essential for developing strategies to diagnose, treat, and prevent COVID-19.

The development of mucosal vaccines against SARS-CoV-2 is a promising approach to induce long-lasting systemic and mucosal immunity. Mucosal vaccination can potentially provide a safe and effective means to protect against SARS-CoV-2, as demonstrated by studies on similar coronaviruses like SARS-CoV and MERS-CoV (4). The gastrointestinal tract, another crucial site for SARS-CoV-2 entry and interaction, underscores the importance of the gut mucosal immune system as a first-line defense. Gastrointestinal symptoms in COVID-19 patients have been associated with worse clinical outcomes, emphasizing the need for vaccines that stimulate mucosal immunity (5).

Current SARS-CoV-2 vaccines primarily induce IgG responses but are limited in their ability to provide mucosal immunity. The role of cytokines such as B-cell activating factor of the TNF family (BAFF) and A proliferation-inducing ligand (APRIL), as well as chemokines like CXCL13, CCL19, and CCL21, is crucial in activating local B-cell responses and antibody production and promoting the formation of inducible bronchus-associated lymphoid tissues (iBALT) (6). These findings suggest that mucosal vaccines may offer more efficient protection against SARS-CoV-2 infection than systemic vaccines. An integrated analysis of SARS-CoV-2 immune responses has revealed distinct systemic and mucosal immune responses during acute infection. The nasopharyngeal microbiome appears to play a role in regulating local and systemic immunity, which in turn determines COVID-19 clinical outcomes (7). In pediatric patients with severe COVID-19 or MIS-C, mucosal neutralizing antibodies in the trachea against circulating SARS-CoV-2 variants were found to be weak, which may have implications for recovery and re-infection (8). Intranasal immunization strategies have shown promise in eliciting mucosal IgA responses, as demonstrated by a study using Lactobacillus plantarum expressing the SARS-CoV-2 spike protein RBD. This approach could serve as a mucosal vaccine candidate against SARS-CoV-2 infection (9). The role of mucosal immune responses in the clearance of SARS-CoV-2 infection and protection against re-infection has been increasingly recognized. Mucosal IgA responses have been detected in infected cases even in the absence of serum antibody responses, with levels correlating strongly with virus neutralization (10). The potential of recombinant probiotics in SARS-CoV2 vaccine development has also been explored, with lactic acid bacteria (LAB) being considered as a practical and safe vaccination approach to halt the virus (11). Intranasal administration of a candidate adenovirus type 5-vectored vaccine (AdCOVID) has been shown to elicit systemic and mucosal immunity and fully protect mice from lethal SARS-CoV-2 challenge (12). This underscores the potential of intranasal vaccination as a strategy to prevent COVID-19 by inducing both systemic and mucosal immunity.

## Immune evasion strategies of coronaviruses at the mucosal immune system

Coronaviruses, such as SARS-CoV, MERS-CoV, and the novel SARS-CoV-2, have greatly endangered global health by causing severe respiratory illnesses and evading the host’s innate immune system. SARS-CoV-2, responsible for the COVID-19 pandemic, has adopted various strategies to overcome antiviral innate immune mechanisms. One such strategy involves suppressing the production of interferon-alpha/beta (IFN-α/β) during the early stages of infection, which is crucial for initiating an immune response against viruses (13). The virus also utilizes mechanisms to exhaust natural killer cell-mediated cytotoxicity, overstimulate the NLRP3 inflammasome, and induce a cytokine storm, resulting in severe inflammation and tissue damage (13).

In comparison, SARS-CoV-2 has been found to impair interferon responses and suppress antigen presentation on both MHC class I and II molecules, which are vital for activating adaptive immune responses. The virus’s ability to induce a cytokine storm is especially detrimental since it can lead to acute respiratory distress syndrome (ARDS) and multiple organ failure (14). The evasion of innate immune responses by SARS-CoV-2 is not only limited to avoiding recognition by host sensors but also includes the impaired production of interferons and antagonizing of the IFN signaling pathways (15). This evasion is coupled with the dysregulated induction of inflammasomes, leading to uncontrolled production of IL-1 family cytokines and pyroptosis, which have been associated with COVID-19 pathogenesis (15). Highly pathogenic human coronaviruses (HP-hCoVs) like SARS-CoV, MERS-CoV, and SARS-CoV-2 have evolved sophisticated interactions with host cells to suppress and evade immune responses. They achieve this by shielding viral RNA from recognition by pattern recognition receptors (PRRs), impairing IFN-I production, and blocking the downstream pathways of IFN-I (16). The immune evasion by SARS-CoV-2 is also linked to the functional exhaustion of lymphocytes and the cytokine storm, which contribute to the immunopathology mechanisms in SARS-CoV-2 infection (17). Immunotherapeutic strategies, such as passive antibody therapy and the use of interferon αβ and IL-6 receptor (IL-6R) inhibitors, are being explored to control the progression of COVID-19 (17). SARS-CoV-2 has shown the ability to evade antibody neutralization, leading to concerns about vaccine effectiveness (18). The virus can impair the interferon antiviral response by targeting mitochondria and can develop tunneling nanotubes to enhance infection and escape immune surveillance (18). Additionally, SARS-CoV-2 is capable of disrupting antigen presentation by reducing MHC-I expression, further evading immune detection (18).

The interaction between endogenous phages and the human immune system has been proposed as a potential avenue for therapeutic development against coronaviruses, including SARS-CoV-2. Phages and phage-based vaccines administered prophylactically could be a valuable strategy in controlling emerging coronavirus infections (19). The evasion of SARS-CoV-2 from the interferon antiviral system is a key aspect of COVID-19 pathogenesis and progression. Various proteins encoded by the virus act as potential interferon antagonists, which present opportunities for the development of antiviral intervention therapies (20). **Table 1** summarizes the different immune evasion strategies employed by coronaviruses.

**Table 1:** Immune evasion strategies of coronaviruses.

Strategy	Mechanism	Consequences	References
Suppression of IFN-α/β Production	- Inhibition of IFN-α/β production during early stages of infection.	- Impaired initiation of antiviral immune response.	(13)
Exhaustion of NK Cell-Mediated Cytotoxicity	- Mechanisms to exhaust natural killer (NK) cell activity.	- Reduced ability to eliminate virus-infected cells.	(13)
Overstimulation of NLRP3 Inflammasome	- Excessive activation of the NLRP3 inflammasome.	- Cytokine storm, severe inflammation, and tissue damage.	(13)
Impaired Interferon Responses	- Targeting mitochondria and disrupting IFN signaling pathways.	- Reduced antiviral activity and increased susceptibility to infection.	(15, 18)
Suppression of Antigen Presentation	- Impaired antigen presentation on MHC class I and II molecules.	- Impaired activation of adaptive immune responses.	(14)
Induction of Cytokine Storm	- Dysregulated production of pro-inflammatory cytokines.	- Acute respiratory distress syndrome (ARDS), multiple organ failure, and severe immunopathology.	(14, 17)
Antibody Neutralization Evasion	- Mutations in viral proteins that reduce antibody binding.	- Reduced vaccine effectiveness and increased risk of re-infection.	(18)
Disruption of Antigen Presentation	- Reduction of MHC-I expression.	- Impaired immune detection and elimination of virus-infected cells.	(18)
Formation of Tunneling Nanotubes	- Creation of tunneling nanotubes between infected and uninfected cells.	- Enhanced virus spread and evasion of immune surveillance.	(18)

## Immune mechanisms and responses of coronaviruses

The immune mechanisms and responses to coronaviruses, particularly SARS-CoV-2, have been a focal point of research due to the global impact of the COVID-19 pandemic (21). A detailed understanding of these responses is crucial for developing effective treatments and vaccines. The innate immune system, as the first line of defense, plays a critical role in the body’s response to SARS-CoV-2 infection. Research has shown that in the respiratory tract of COVID-19 patients, there is a heightened expression of proinflammatory genes, particularly chemokines, leading to hypercytokinemia. This suggests that SARS-CoV-2 infection triggers a robust inflammatory response, which is different from the inadequate interferon (IFN) responses observed in SARS-CoV infections. The study also noted an increase in activated dendritic cells and neutrophils, indicating a vigorous innate immune response (21).

Coronaviruses have developed sophisticated mechanisms to evade the innate immune system. SARS-CoV-2, for instance, can induce a cytokine storm, impair interferon responses, and suppress antigen presentation on MHC class I and II molecules. Understanding these evasion strategies is essential for grasping the pathogenesis of the virus and for the development of vaccines and immunotherapeutic (14). The innate immune system detects viral components through various cellular receptors, leading to the production of interferons and cytokines that help eliminate the virus. However, coronaviruses have evolved to evade these antiviral immune responses, which may contribute to their ability to infect multiple species and cause diseases like SARS, MERS, and COVID-19 (22). The interplay between innate and adaptive immunity is crucial for the clearance of coronavirus infections (23). However, uncontrolled immune responses can lead to acute lung injury and significant immunopathology. A profound understanding of the interaction between coronaviruses and the immune system is vital for treatment strategies (23).

The pathogenesis of coronaviruses influences innate immune responses, inflammasome activation, inflammatory cell death pathways, and cytokine secretion. Studies on SARS-CoV and MERS-CoV have laid the groundwork for understanding the innate immunity against SARS-CoV-2 (24). In severe COVID-19 cases, there is a robust expansion of the innate immune response, with a marked reduction in several lymphoid cell types and an increase in heterogeneity within plasmablasts. The immune remodeling in severe cases is distinct from non-severe cases, with an increased aggregation potential of myeloid subsets, particularly monocytes (25). The interaction between SARS-CoV-2 and human macrophages triggers a strong innate immune response characterized by a pro-inflammatory storm, which can lead to end-organ tissue damage. This response is marked by robust cytokine and chemokine expression but attenuated type I interferon activity (26).

Coronavirus receptors on host cells not only facilitate viral entry but also modulate immune responses (27). These receptors play a role in the hyperinflammatory response observed in severe infections, and understanding their function could provide insights into the pathological responses to coronavirus infections.

The immunopathogenesis of COVID-19 involves both antiviral immune responses and uncontrolled inflammatory responses. The immune abnormalities induced by SARS-CoV-2 can lead to severe multiple organ dysfunction. Managing these immune responses, by enhancing antiviral immunity and inhibiting systemic inflammation, may be key to successful treatment (28). The adaptive immune response, particularly B and T cell activity, is crucial for viral clearance. SARS-CoV-2 employs mechanisms to counter the adaptive immune response, such as T cell depletion and exhaustion, which are linked to disease severity. Understanding the B and T cell responses in SARS-CoV-2 pathogenesis is vital for addressing the challenges of reinfection and developing long-term immunity (29).

## Vaccines and mucosal immunity of coronaviruses

The emergence of SARS-CoV-2 and the ongoing COVID-19 pandemic have underscored the importance of understanding and enhancing mucosal immunity through vaccination. The mucosal immune system, which includes the largest component of the entire immune system, plays a critical role in providing protection at the main sites of infectious threat, such as the respiratory and gastrointestinal mucosae (3, 5). As SARS-CoV-2 primarily infects the upper respiratory tract, the induction and effector phases of the immune response are initiated at these mucosal surfaces (3). Mucosal vaccines aim to elicit an immune response at the site of virus entry, thereby potentially preventing infection and transmission. Studies have shown that mucosal vaccination can induce long-lasting systemic and mucosal immunity, which is crucial for protection against respiratory viruses like SARS-CoV-2 (4). The development of mucosal vaccines against SARS-CoV-2 is therefore a promising approach to combat the pandemic (4, 30).

The mucosal and serological immune responses to SARS-CoV-2 vaccines have been extensively studied. mRNA vaccines like Comirnaty have been found to elicit specific immunoglobulin responses in the nasal mucosa (31). This may offer an additional layer of protection compared to inactivated virus vaccines such as CoronaVac. Mucosal immunity, particularly the presence of IgA antibodies, is believed to play a beneficial role in limiting viral replication and clearing the virus (10). In fact, a strong mucosal immune response may contribute to the early restriction of SARS-CoV-2 infection (10). It is also important to note that the mucosal immune system of the gastrointestinal tract serves as a critical defense against SARS-CoV-2 (5). Poor clinical outcomes in COVID-19 patients with gastrointestinal involvement have been reported (5). Therefore, vaccines that can stimulate mucosal immune responses in the gut may offer advantages in preventing and controlling the infection.

Several strategies have been explored to enhance mucosal immune responses, including the use of recombinant Lactobacillus plantarum expressing the receptor-binding domain (RBD) of the SARS-CoV-2 spike protein, which has shown promise in eliciting mucosal IgA antibodies in the respiratory and intestinal tracts in mice (12). Similarly, intranasal administration of an adenovirus-vectored vaccine encoding the RBD of the SARS-CoV-2 spike protein resulted in strong mucosal and systemic immunity, providing complete protection in mice (12). The role of cytokines and chemokines, such as BAFF, APRIL, CXCL13, CCL19, and CCL21, in the activation of local B-cell responses and antibody production, as well as the formation of inducible bronchus-associated lymphoid tissues (iBALT), has been reviewed, suggesting that mucosal vaccines may offer more efficient protection against SARS-CoV-2 infection (6). Furthermore, a mucosal TLR2-activating protein-based vaccine has been shown to induce potent pulmonary immunity and protection against SARS-CoV-2 in mice, highlighting the potential of mucosal vaccination strategies (32). **Table 2** outlines various strategies currently being explored to enhance mucosal immunity against coronaviruses.

**Table 2:** Strategies for enhancing mucosal immunity against coronaviruses.

Strategy	Mechanism of Action	Benefits	Challenges and Considerations	References
Mucosal Vaccination	- Stimulates immune response at the site of virus entry (respiratory and gastrointestinal mucosa).	- Induction of long-lasting systemic and mucosal immunity.	- Development of effective delivery systems.	(4, 30)
	- Primarily elicits IgA antibody response.	- IgA antibodies play a crucial role in neutralizing the virus and preventing infection.		(3)
Intranasal Vaccination	- Directly targets the nasal mucosa, the initial barrier against SARS-CoV-2 entry.	- Induces strong mucosal and systemic immunity, including IgA antibodies, neutralizing antibodies, and T cell responses.	- Optimization of vaccine formulation and dosage.	(12)
Recombinant Probiotics	- Utilize bacteria (e.g., Lactobacillus plantarum) expressing viral proteins to stimulate mucosal immune responses.	- Safe and practical approach to vaccine delivery.	- Ensuring stability and efficacy of the probiotic.	(9, 11)
Cytokines and Chemokines	- BAFF, APRIL, CXCL13, CCL19, CCL21 enhance local B-cell responses, antibody production, and formation of inducible bronchus-associated lymphoid tissues (iBALT).	- Potential for more efficient protection against SARS-CoV-2 infection compared to systemic vaccines.	- Balancing immune activation to avoid excessive inflammation.	(6)
TLR2-Activating Protein-Based Vaccine	- Activates TLR2 receptors to induce potent pulmonary immunity.	- Strong induction of mucosal and systemic immunity against SARS-CoV-2.	- Need for further research to optimize vaccine design.	(32)
Immunovac-VP-4 Vaccine in Conjunction with Basic Therapy	- Stimulates mucosal immune responses in the upper respiratory tract.	- Increases levels of secretory IgA (sIgA) at the mucosal surfaces, potentially aiding in viral clearance and recovery.	- Further research needed to assess long-term efficacy.	(33)
Plant Bioactive Molecules and Glycan-Binding Lectins	- Target viral proteins for activation of mucosal immune responses.	- May offer a novel approach to stimulate mucosal immunity and provide protection.	- Identification and optimization of suitable molecules.	(34)

## Significance of mucosal immunity in defending against coronaviruses

Most current SARS-CoV-2 vaccines induce specific IgG responses but provide limited mucosal immunity. Novel vaccine development considerations include the use of cytokines such as BAFF and APRIL, and chemokines like CXCL13, CCL19, and CCL21 to enhance local B-cell responses and antibody production, potentially offering more efficient protection against SARS-CoV-2 infection (6). Mucosal immunity plays a key role in the early restriction of viral replication and the clearance of SARS-CoV-2. The angiotensin-converting enzyme 2 (ACE2) receptor, which is the cellular entry point for the virus, is most highly expressed in the upper respiratory tract. Mucosal IgA responses have been detected in infected cases in the absence of serum antibody responses, with levels correlating strongly with virus neutralization (10). The role of mucosal immunity in SARS-CoV-2 vaccine development is further emphasized by the potential use of recombinant probiotics. These can act as vaccine carriers due to their immunomodulatory features and natural adjuvanticity, offering a practical and safe approach to halt the virus (11). Distinct systemic and mucosal immune responses are observed during acute SARS-CoV-2 infection. Nasopharyngeal viral load correlates with humoral responses but inversely with interferon responses, which are associated with protective microbial communities. This compartmentalization of immune responses highlights the role of the nasopharyngeal microbiome in regulating local and systemic immunity that determines COVID-19 clinical outcomes (7).

IgG Fc-binding protein (Fcγbp), which is secreted by airway mucin-producing cells, has been found to play a protective role against SARS-CoV-2 infection. Fcγbp can capture cross-reactive IgG antibodies that are bound to the virus and eliminate them from the mucosal surface. This mechanism potentially reduces viral loads and prevents excessive immune reactions (35). When it comes to activating immune responses against pathogens entering through mucosal surfaces, activating mucosal immunity is considered a more favorable approach. Mucosal vaccines can stimulate robust systemic humoral immunity, which is essential for neutralizing any virus particles that evade the initial immune response (34).

In the context of emerging coronaviruses, the presence of cross-reactive immunity is an important consideration. Alturaiki (36) propose the development of a mucosal universal vaccine that can provide long-term protection against current and emerging coronaviruses by leveraging cross-reactive immunity. Such a vaccine would enhance the local mucosal adaptive response, inhibit viral replication, and promote viral clearance.

Moreover, intranasal vaccination has been demonstrated as an effective strategy for preventing COVID-19. King et al. (12) found that a single intranasal dose of the AdCOVID vaccine candidate induced a robust immune response, including the production of mucosal IgA, serum neutralizing antibodies, and T cells with a Th1-like cytokine expression profile. In a mouse model, this vaccination approach provided complete protection against lethal SARS-CoV-2 challenge.

## Role of mucosal immunity in COVID-19

The significance of mucosal immunity in the context of COVID-19 infection is often overlooked, even though it plays a crucial role in defending against SARS-CoV-2. The mucosal immune system, which is the largest component of the overall immune system, provides protection at the main sites of infection, such as the mucosae (3). While SARS-CoV-2 primarily infects the upper respiratory tract, the interaction with the immune system occurs predominantly at the respiratory mucosal surfaces. Unfortunately, most research has focused on serum antibodies and systemic cell-mediated immunity, neglecting the potential importance of mucosal immunity and secretory IgA antibodies in understanding asymptomatic and mild infections (3).

To address this gap, intranasal vaccination has emerged as an attractive strategy for preventing COVID-19. Unlike current intramuscular vaccines that primarily elicit systemic immunity, intranasal vaccination targets the nasal mucosa, which is the initial barrier against SARS-CoV-2 entry (12). A study on a single intranasal dose of an adenovirus-vectored vaccine encoding the SARS-CoV-2 spike protein receptor-binding domain (AdCOVID) has shown promising results. This vaccination approach induced strong mucosal IgA responses, serum neutralizing antibodies, and T cell responses, leading to complete protection against lethal SARS-CoV-2 challenge in mice (12).

The expression of the angiotensin-converting enzyme 2 (ACE2) receptor, which is the cellular entry point for SARS-CoV-2, is highest in the upper respiratory tract (10). Studies have found that mucosal IgA responses can be detected in the absence of serum antibody responses, with mucosal antibody levels strongly correlating with virus neutralization (10). This suggests that mucosal immune responses play a key role in the early restriction of viral replication and clearance of SARS-CoV-2 (10). The gastrointestinal tract may also serve as an entry or interaction site for SARS-CoV-2, making the gut mucosal immune system a critical first-line defense (5). Gastrointestinal involvement in COVID-19 patients has been associated with worse clinical outcomes, highlighting the importance of understanding the interactions between the virus and the immune cells and molecules in the mucosa. Vaccines that stimulate mucosal immunity against the virus may offer more advantages than others (5). The development of novel COVID-19 mucosal vaccines is being considered, as most vaccines induce specific IgG responses but provide limited mucosal immunity (6). Factors such as cytokine B-cell activation factor (BAFF), A proliferation-inducing ligand (APRIL), and homeostatic chemokines could play key roles in activating local B-cell responses and antibody production, as well as promoting the formation of inducible bronchus-associated lymphoid tissues (iBALT) (6). The nasal immune cell populations show prolonged activation following COVID-19, with the development of tissue-resident SARS-CoV-2 specific CD8 T cell responses (37).

This indicates that COVID-19 has both transient and long-term effects on the immune system in the upper airway (37). Secretory immunoglobulin A (sIgA) is a significant indicator of the mucosal immune response of the respiratory tract (38). sIgA antibodies are effective against various pathogens, including SARS-CoV-2, and are detected in patients with COVID-19 early after symptom onset (38). Intranasal immunization has been shown to induce the predominant production of sIgA in the respiratory tract (38). The potential of mucosal immunity and recombinant probiotics in SARS-CoV2 vaccine development is being explored (11). Mucosal vaccination could elicit mucosal and systemic immune responses simultaneously, with recombinant probiotics, particularly lactic acid bacteria (LAB), being considered as a practical and safe vaccination approach (11). Activation of mucosal immunity is a novel prophylactic and therapeutic strategy in combating COVID-19. Mucosal vaccines could generate strong systemic humoral immunity and neutralize any virus particles that evade the primary immune response (34). The use of plant bioactive molecules and glycan-binding lectins against viral proteins for targeted activation of mucosal immune response is also being considered (34). Finally, the role of mucosal immunity in the treatment of COVID-19 patients is highlighted by a study assessing the concentrations of sIgA in the upper respiratory tract (33). The use of the bacterial vaccine Immunovac-VP-4 in combination with basic therapy was associated with an increase in sIgA levels at the mucous surfaces of the respiratory tract (33).

## Research and clinical relevance of coronaviruses

The recent outbreak of coronavirus disease (COVID-19) has sparked a significant amount of research aimed at understanding the epidemiology, clinical manifestations, diagnosis, prevention, control, and therapeutic interventions for coronaviruses, particularly the novel severe acute respiratory syndrome coronavirus 2 (SARS-CoV-2) (39). This review aims to synthesize the current state of knowledge based on recent studies. The COVID-19 pandemic, caused by SARS-CoV-2, originated in Wuhan, China, and has since spread globally, leading to an unprecedented public health crisis (39–41). The rapid spread and high pathogenicity of the virus have necessitated a surge in research efforts to understand the virus’s epidemiology, causes, and clinical manifestations (39). Studies have shown that the virus likely originated from a seafood market in Wuhan, with bats being the possible primary reservoir, although the exact intermediate source remains unidentified (42). Human-to-human transmission has been confirmed, and the virus uses the angiotensin-converting enzyme 2 (ACE2) receptor for cell entry, like SARS-CoV (40, 42).

Clinical manifestations of COVID-19 range from mild symptoms like fever, cough, and fatigue to severe complications such as pneumonia, acute respiratory distress syndrome (ARDS), and multi-organ failure (39, 43). The disease has a higher impact on the elderly and individuals with underlying health conditions (40, 43). Despite the urgent need, there are currently no clinically approved vaccines or specific antiviral drugs for COVID-19. However, several therapeutic interventions, including repurposed drugs and monoclonal antibody therapy, are under investigation (44, 45). Preventive measures such as masks, hand hygiene, social distancing, case detection, contact tracing, and quarantine have been crucial in controlling the spread of the virus (39). The psychological, behavioral, and interpersonal effects of the pandemic and the associated preventive measures have also been significant, affecting billions of people worldwide and posing challenges to mental health and healthcare systems (46).

Antiviral drugs that inhibit viral proliferation, drugs that modulate the host’s immune response, and plant-derived compounds with antiviral activity have been identified as potential therapeutic options for COVID-19 (45). Ongoing clinical trials are evaluating the efficacy and safety of these treatments (45). The immune response plays a critical role in the outcome of coronavirus infections. An appropriate immune response can control and eliminate the infection, while an excessive or maladjusted response can lead to immunopathology and lung inflammation (47). To develop strategies to mitigate lung inflammation and other complications, it is crucial to understand the interaction between coronaviruses and the host’s immune system (47).

## Conclusion

A thorough understanding of the immune response to coronaviruses is crucial for the development of effective vaccines and therapies for COVID-19. Notably, mucosal immunity plays a significant role in defending against SARS-CoV-2, and investigating its mechanisms is essential for comprehensive protection against the virus. Exploring the use of mucosal vaccines and intranasal immunization strategies shows promise in achieving this goal. Additionally, understanding how coronaviruses like SARS-CoV-2 evade the immune system is crucial in developing effective interventions, as their ability to suppress immune responses and induce hyperinflammatory reactions contributes to the severity of COVID-19. Ongoing research in these areas is critical for the development of therapeutic and preventive strategies. Furthermore, the development of mucosal vaccines is a promising approach to induce protective immunity and prevent viral transmission and infection. Research in this field has the potential to play a pivotal role in controlling pandemics and preventing future outbreaks.
